# Congenital Hypothyroidism: An Unusual Combination of Biochemical Abnormalities

**DOI:** 10.1155/2016/2678578

**Published:** 2016-11-22

**Authors:** Ruchi Mantri, S. B. Bavdekar, Sushma U. Save

**Affiliations:** Department of Pediatrics, TN Medical College and BYL Nair Charitable Hospital, Dr. AL Nair Road, Mumbai Central, Mumbai 400008, India

## Abstract

A forty-five-day-old female infant presented with prolonged jaundice with clinical features suggestive of congenital hypothyroidism (CHT). On investigations, the infant was noted to have indirect hyperbilirubinemia (13.8 mg/dl) with increased levels of AST (298 IU/dl) and ALT (174 IU/dl) in the serum. The child had low levels of free T_3_ (<1 pg/ml) and free T_4_ (0.4 ng/dl) secondary to thyroid agenesis detected on radionuclide scan and ultrasonography of the neck and raised levels of TSH (>500 microIU/ml) in the serum. The combination of indirect hyperbilirubinemia and raised levels of hepatic transaminases has not been reported in babies with CHT. Following institution of oral thyroxin therapy, the serum bilirubin levels ameliorated (2.9 mg/dl) considerably by 15 days of therapy and the serum levels of AST (40 IU/dl) and ALT (20 IU/dl) got normalized. The case demonstrates that raised levels of hepatic transaminases can occur in infants with CHT and these can resolve just with thyroxin therapy, obviating the need for extensive investigative laboratory work-up.

## 1. Introduction

As neonatal screening program is not implemented in several resource-poor settings, babies with congenital hypothyroidism (CHT) present with a spectrum of clinical manifestations of varying severity [1]. Pathological neonatal jaundice is one such nonspecific manifestation of CHT. The hyperbilirubinemia associated with CHT can be conjugated or unconjugated and a few cases of conjugated hyperbilirubinemia with raised levels of transaminases have been described in literature [2]. Through this communication, we intend to describe a case of CHT with indirect hyperbilirubinemia and persistently raised levels of hepatic transaminases, a combination not hitherto described.

## 2. Case Description

A 45-day-old exclusively breastfed female infant born at term (birth weight: 3033 g) vaginally to a healthy mother, presented with jaundice noticed since the sixth day of life. The jaundice that was not associated with clay colored stools or high colored urine increased progressively and was associated with constipation and excessive sleepiness. There was no history of hypothyroidism in the mother nor had she consumed any drugs other than hematinics during the ante-natal period. Both mother's and baby's blood group was O positive. The mother was negative for hepatitis B surface antigen (HBsAg) during pregnancy. On examination, the infant was hemodynamically stable (pulse 122/min, BP: 68/42 mm Hg, no cold extremities), weighed 3.5 kg (at third percentile, WHO Child Growth Standards), and had a length of 52 cm (between 3rd and 50th percentile, WHO Child Growth Standards; upper : lower segment ratio: 1.6). She had coarse facies, open posterior fontanel (2 mm × 2 mm), a large open anterior fontanel (3 × 3 cm), macroglossia, hoarse cry, icterus involving the whole body including palms and soles, reducible umbilical hernia (ring 3 × 3 cm), and dry skin. The baby had no goiter or neck swelling. The baby's neurological examination was normal. She had normal tone and there was no delayed relaxation of tendon reflexes. Liver and spleen were not palpable on abdominal examination.

The clinical diagnosis of neonatal hyperbilirubinemia in a child with CHT was confirmed with laboratory investigations (Table 1; Hb: 11.7 gm/dl, no evidence of hemolysis on peripheral smear, reticulocyte count: less than 1% and Coomb's test: negative). The levels of SGOT (AST) and SGPT (ALT) were raised but there was no biochemical evidence of hepatic biosynthetic dysfunction (normal levels of serum albumin and prothrombin time). Proximal tibial/distal femoral epiphyses were seen on the radiograph of the lower limbs. Ultrasound of the neck showed absence of thyroid gland at the usual site and there was no evidence of ectopic thyroid tissue in the neck. Thyroid scan (Tc-99 thyroid scintigraphy) showed no evidence of functioning thyroid tissue in the thyroid bed or head and neck region. These investigations confirmed that thyroid gland agenesis is the cause of congenital hypothyroidism. Two-dimensional echocardiography did not reveal any abnormality. Abdominal ultrasound showed presence of hepatosplenomegaly (with normal echotexture), which resolved by day 74. The child was treated with L-thyroxine (10–15 microgm/kg/d) which resulted in decrease in jaundice, lowered levels of serum bilirubin and TSH, and normalization of T_3_, T_4_, AST, and ALT levels in the serum by 15th day of therapy (Table 1).

## 3. Discussion

This communication describes an infant with congenital hypothyroidism with prolonged indirect jaundice and persistently increased levels of hepatic transaminases (AST and ALT). When treated with L-thyroxin, most of the biochemical abnormalities normalized within 15 days of therapy and TSH levels decreased significantly. The association between congenital hypothyroidism (CHT) and neonatal (conjugated or unconjugated) hyperbilirubinemia is long established. A few cases of CHT with unconjugated hyperbilirubinemia have raised levels of hepatic transaminases in the blood, which resolve following treatment with thyroxin [2].

Despite extensive literature search, we could not locate any case of CHT with indirect hyperbilirubinemia and raised levels of hepatic transaminases. When associated with conjugated hyperbilirubinemia, the raised transaminase levels in infants with CHT are attributed to cholestasis. Our case suggests that raised transaminase levels might be occurring independent of hepatic involvement. In this context, it is worthwhile remembering that although liver is an overwhelmingly predominant source of transaminases, there are other sources of AST (cardiac and skeletal muscles, kidneys, brain, pancreas, lungs, and erythrocytes) and ALT (skeletal muscle and kidneys). It is also well-known that myriad myopathies are associated with raised levels of AST/ALT and that these abnormal levels could precede clinical manifestations of myopathy by several weeks [3]. Thyroxin deficiency is known to be associated with myopathy [4] involving the skeletal and cardiac muscle. Hence, we think that the raised levels of AST and ALT seen in the infant were due to hypothyroidism-associated myopathy. The infant's two-dimensional echocardiography did not reveal any abnormality. However, it is possible that a part of the raised levels of AST could be attributed to subclinical cellular-level cardiac involvement. It is true that some asymptomatic infants and children have transiently raised serum levels of AST and ALT, which resolve over a period of time [5, 6]. We think that the infant reported here does not belong to this group of infants. This is also not a chance occurrence, as the biochemical abnormalities began normalizing within days of institution of thyroxin therapy. This temporal relationship between the administration of thyroxin and beginning of resolution of manifestations cannot be neglected.

This case report has practical implications: the case indicates that presence of raised transaminase levels in infants with CHT does not necessarily indicate presence of liver diseases and hence there is no need to subject such infants to invasive diagnostic tests. We also hope that this report will motivate others to report such cases in their practice, which would provide credence to the notion of this being a real association.

## Figures and Tables

**Table 1:** Results of serial biochemical investigations.

Day of life	Reference value	44	51	52	55	59	70
Day following initiation of therapy		−11	−4	−3	0	4	15
Serum bilirubin (total)	<1.2 mg/dl	14.9	16	15.2	14.6	16.3	3.8
Serum bilirubin (direct)	<0.2 mg/dl	1.1	1.3	1.3	1.3	1.1	0.9
Serum bilirubin (indirect)	≤1.0 mg/dl	13.8	14.7	13.9	13.3	15.2	2.9
Serum protein, total	5.1–7.3 g/dl	—	5.6	5.3	5.6	5.7	6.0
Serum albumin	2.2–4.8	—	3.4	3.3	3.4	3.6	4.1
SGOT (AST)	9–80 u/l	—	298	278	225	220	40
SGPT (ALT)	13–45 u/l	—	174	157	148	135	20
Serum alkaline phosphatase	150–420 u/l	—	239	213	226	221	247
Serum free T_3_	0.91–4.4 pg/ml	—	—	—	<1	—	4.1
Serum free T_4_	0.8–2.0 ng/dl	—	—	—	<0.4	—	1.4
TSH	0.8–8.2 microIU/ml	—	—	—	500	—	37.8
